# Optimising Prescription of Treatment In older patients with Mild hypertension at Increased risk of Serious adverse Events (OPTIMISE2): protocol for a primary care based, open-label, randomised controlled non-inferiority trial

**DOI:** 10.1186/s13063-026-09508-7

**Published:** 2026-02-14

**Authors:** Melanie Carr, Nicola Kenealy, Ly-Mee Yu, Joanna Moschandreas, F. D. Richard Hobbs, Simon de Lusignan, Gary Ford, Stavros Petrou, Milensu Shanyinde, David McCartney, Andrew Clegg, Jonathan Mant, Rupert Payne, Marney Williams, Margaret Ogden, Richard J. McManus, James P. Sheppard

**Affiliations:** 1 Nuffield Department of Primary Care Health Sciences, University of Oxford, Oxford, UK; 2 Oxford Institute of Digital Health, Oxford, UK; 3 Radcliffe Department of Medicine, University of Oxford, Oxford, UK; 4 University of Leeds, Leeds, UK; 5 Primary Care Unit, Department of Public Health & Primary Care, University of Cambridge, Cambridge, UK; 6 University of Exeter, Exeter, UK; 7 Patient and Public Contributor, London, UK

**Keywords:** Randomised controlled trial, Blood pressure, Deprescribing, Medication discontinuation, Serious adverse events, Primary care, Aged, Frailty, Mortality, Cardiovascular disease

## Abstract

**Background:**

Antihypertensive treatment is effective at reducing the risk of cardiovascular disease, but is associated with adverse events, particularly in older patients with frailty. As a result, deprescribing antihypertensive medications is recommended in some clinical guidelines despite limited evidence from a few small randomised controlled trials. The aim of the OPTIMISE2 trial is to examine the safety, efficacy and cost-effectiveness of deprescribing antihypertensive treatment in older adults with controlled systolic blood pressure, who are at higher risk of adverse events.

**Methods:**

The OPTIMISE2 trial aims to enrol 3014 participants into the trial and actively follow them up for 1 year. Participants are aged 75 years and above and taking two or more blood pressure lowering drugs, with controlled blood pressure readings (systolic blood pressure < 140 mmHg if aged 75–79 years or < 150 mmHg if aged 80+ years), frail and/or at a higher risk of serious drug-related side effects of hypotension, syncope and falls. The trial randomises (1:1) participants to either step-down medication reduction (withdrawal of one antihypertensive medication at a time, at 4-week intervals with regular monitoring of blood pressure) or usual care (no medication reduction mandated). The choice of medications to withdraw is at the discretion of participating general practitioners or prescribers. The primary outcome is emergency hospitalisation or death within 1 year of randomisation. The primary objective is to determine whether antihypertensive deprescribing is non-inferior to usual care, with up to a 5% difference in the percentage of patients with an emergency hospitalisation/death. The study uses a within-trial economic evaluation and decision-analytic modelling to estimate the cost-effectiveness of deprescribing antihypertensive treatment.

**Discussion:**

It is expected that these findings will inform clinical guidelines and practice about deprescribing of antihypertensive medications in older adults with frailty, who have controlled systolic blood pressure but are at higher risk of adverse events.

**Trial registration:**

ISRCTN18030225. Registered on 19/09/2023.

Secondary Identifying Numbers: University of Oxford Sponsor Protocol Number 16667. IRAS 1006598. CPMS 56390.

**Supplementary Information:**

The online version contains supplementary material available at 10.1186/s13063-026-09508-7.

## Background

In England, approximately 1.5 million adults aged over 75 are prescribed five or more medications [1, 2] to treat their long-term conditions, a situation described as polypharmacy. Inappropriate polypharmacy (where patients are prescribed too many medications for the conditions present) [3] is associated with reduced independence and quality of life, and an increased likelihood of hospital admissions for drug-related adverse events [4, 5]. One approach to managing polypharmacy is to deprescribe medications which may no longer provide benefit or where the chance of adverse events is high. Deprescribing is the process of withdrawing or reducing the use of medications that are considered inappropriate or unnecessary for a particular patient, supervised by a health care professional, to improve health outcome [6].

Drugs to manage blood pressure are highly effective at reducing the risk of cardiovascular disease [7, 8] and are the most frequently prescribed medication in patients with polypharmacy [1]. However, they are also associated with potential harm [7], increasing the risk of hypotension, syncope, acute kidney injury and electrolyte abnormalities, and these adverse effects are more likely in older patients and those with increasing frailty [7, 8]. As a result, clinical judgement is encouraged when prescribing antihypertensive treatment in older adults with frailty [9], and deprescribing antihypertensive medications is now recommended in some clinical guidelines [10] despite limited evidence from only a few small randomised controlled trials.

The University of Oxford team previously completed the largest trial of blood pressure medication reduction to date, enrolling 569 participants and following them up for 12 weeks. This study demonstrated that deprescribing antihypertensives was non-inferior to usual care with regard to blood pressure control (RR 0.98, 95% CI 0.92 to ∞), albeit with an increase in mean systolic blood pressure of 3 mm Hg at 12 weeks [11]. Long-term follow-up via participants’ electronic health records found no evidence of an association between deprescribing and all-cause hospitalisation or mortality [12]; however, this trial was not powered to determine the long-term effects of deprescribing on clinical outcomes. Therefore, it is not currently known if deprescribing is safe or effective in the longer term. This trial examines the effect of antihypertensive deprescribing on clinical outcomes related to benefit and harm, health-related quality of life and costs to the NHS.

### Trial design

This trial uses an individually randomised, controlled, non-inferiority design, based on the trial design successfully tested and implemented in the previous OPTiMISE trial [11, 13, 14]. It aims to establish whether deprescribing antihypertensives is safe in older people, examining the following research questions:

1. What is the effect of deprescribing blood pressure lowering drugs on hospital admissions and death?
2. What is the effect of deprescribing on costs to the NHS and health-related quality of life?

The trial compares step-down medication reduction (withdrawal of one antihypertensive medication at a time, at 4-week intervals, with monitoring of blood pressure at 4 weeks’ visit following each withdrawal) with usual care (no medication reduction mandated). Participants are expected to attend a baseline visit and a 4-week safety visit per antihypertensive deprescribed. Subsequent follow-up data is collected via routine electronic health records and participant questionnaires sent at 1-year post-randomisation. The primary outcome is emergency hospital admission and all-cause death at 1 year. The trial team will also perform a cost-effectiveness analysis to assess the short and long-term impacts of deprescribing antihypertensives.

This trial is undertaken in a primary care setting, within approximately 200 general practices across all regions of the NIHR Research Delivery Network in England. The primary care setting enables all potentially eligible participants to be identified and approached, and permits examination of the intervention, in the setting in which it will be delivered in routine clinical practice.

### Trial outcomes

The primary outcome is all-cause emergency hospitalisation or death during the 12 months following randomisation. This is determined using health record data obtained from NHS England using ‘method of admission’ codes 21–25 and 28 (admission for at least 1 day overnight) or death, and from practice-reported serious adverse events. Secondary outcomes are major cardiovascular events, a cardiovascular death, all-cause death, all-cause emergency hospitalisation, all-cause hospitalisation, acute myocardial infarction, stroke, heart failure, serious falls, fractures, hypotension, syncope, dementia, a serious adverse event, a non-serious adverse event, an adverse drug withdrawal event, or admission to a nursing home or care facility between groups. Further secondary outcomes are to determine the difference in average treatment burden, average medication burden, and average antihypertensive medication between groups and those in the intervention arm who maintain medication reduction through to follow-up. The trial also measures the difference in average blood pressure and the percentage of participants experiencing symptoms such as dizziness, fatigue and ankle swelling, using the Beliefs about Medicines Questionnaire (BMQ) General [15]. The EuroQol 5-Dimension 5-Level Questionnaire (EQ-5D-5L) [16] index is used to determine the difference in health-related quality of life between randomised groups and the Katz Activities of Daily Living – Life Ability Questionnaire [17] is used to determine the percentage of participants with a physical disability between groups.

### Recruitment

The population of interest is adults aged ≥ 75 years with controlled systolic blood pressure (< 140 mmHg if aged 75–79 years or < 150 mmHg if aged ≥ 80 years), receiving two or more antihypertensive medications and at higher risk of serious adverse events. Potentially eligible participants are identified through a specially designed search tool incorporating the trial inclusion and exclusion criteria, run through general practice electronic health records.

#### Inclusion criteria

- Willing and able to give informed consent for participation in the trial (or with personal legal representative consent)
- Willing and able to report any safety concerns or with a suitable carer able to report these if unable
- Registered at either a practice using electronic health record systems (e.g. EMIS or SystmOne) or contributing to or willing to contribute to The Oxford Clinical Informatics Digital Hub ORCHID [18].
- Aged 75 years or above at recruitment.
- Controlled systolic blood pressure, defined (in accordance with NICE 2019 guidelines) [9] as less than 140 mmHg (if aged 75–79 years) or less than 150 mmHg (if aged 80 years or above). Systolic blood pressure level will be based on screening measurements taken at baseline (mean of the 2nd and 3rd readings taken in a standardised manner) [19] or from patient records if a face-to-face baseline visit is not possible.
- Prescribed two or more antihypertensive medications for at least 12 months prior to trial entry. Antihypertensive medications defined as any ACE inhibitor, angiotensin II receptor blocker, calcium channel blocker, thiazide and thiazide-like diuretic (including loop diuretics), potassium-sparing diuretic, alpha-blocker, beta-blocker, vasodilator antihypertensives, centrally acting antihypertensives, direct renin inhibitors, adrenergic neurone blocking drugs.
- Stable dose of antihypertensive medications for at least 4 weeks prior to trial entry.
- Moderate or severe frailty (defined by an eFI score ≥ 0.20) [20] and/or high risk (> 5%) of hypotension, syncope or falls in the next 5 years, based on STRATIFY risk prediction algorithms [21, 22] applied to an individual’s electronic health record.

#### Exclusion criteria

- Heart failure due to left ventricular systolic dysfunction (LVSD) prescribed only ACE inhibitors/angiotensin II receptor blockers and/or beta-blockers and/or spironolactone (removing any of which would be contraindicated).
- Heart failure diagnosis without a coded echocardiogram (might have undiagnosed LVSD and a compelling need for ACEI/angiotensin II receptor blocker and beta-blockers).
- Suffered a myocardial infarction or stroke within the past 6 months.
- Secondary hypertension or previous accelerated or malignant hypertension.
- Lacking capacity to give consent and without a Personal Legal Representative present at the point of screening.
- Investigator deems that there is a compelling indication for medication continuation.
- Participating in any other randomised controlled trial of drug treatment or interventional medical devices in the past 4 weeks (can be re-invited subsequently).

**Fig. 1:**
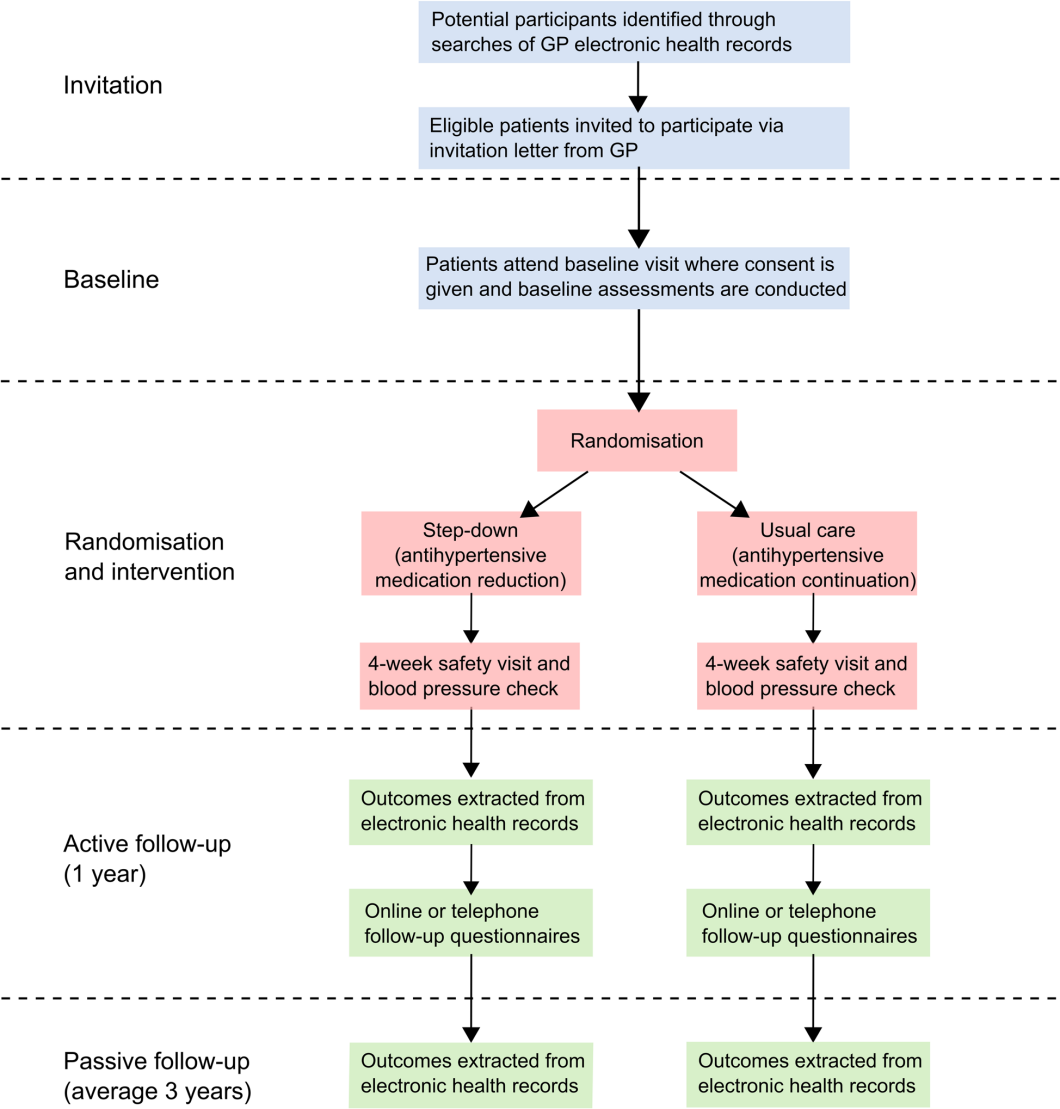
Trial flow diagram. GP, general practitioner; the 4-week safety visit is repeated each time a medication is withdrawn

The participant journey through the trial is described in Fig. 1. Those potential participants who respond to an invitation attend a baseline visit at their general practice. At this visit, the general practitioner (GP) (or qualified and appropriately trained health professional responsible for hypertension care or medicines management) explains the trial and gives time to answer any questions. Following this, if they are happy to proceed, they will be asked to give informed consent. The trial aims to include vulnerable individuals, including those with dementia and those lacking capacity to consent for themselves. In these instances, consent from an appropriate Personal Legal Representative is requested.

### Baseline assessments

After consent, participants undergo baseline procedures with a research nurse, or other appropriately trained and delegated member of the team, including measurement of clinic blood pressure readings. Blood pressure is taken after participants have been seated for 5 min of rest using a clinically validated blood pressure monitor, and the mean of the 2nd and 3rd readings is used the define trial eligibility. To test for orthostatic hypotension, two further readings are taken in the standing position, immediately, and after 3 min [23]. Once eligibility is confirmed, the participant then completes the trial questionnaires including the Illness Perception Questionnaire (symptom list) [24], Multimorbidity Treatment Burden Questionnaire [25], Katz Activities of Daily Living (ADL) - Life Ability Questionnaire [17], and Beliefs about Medicines Questionnaire (BMQ) General [15] and the EQ-5D-5L [16] which will give an interpretation of quality of life. Also included is an adapted variant of the Client Service Receipt Inventory [26] to measure health economic burden, the Free-Cog [27] to assess cognition, and the Attitudes Towards Deprescribing Questionnaire [28] to determine patient perception of medication reduction. Once these questionnaires have been completed to the best of their ability, they are then randomised into the trial by the person performing the baseline procedures. Stratified randomisation is used to individually allocate participants (1:1) to one of the two trial groups using a fully validated web-based randomisation system (Sortition) with stratification factors age (i.e. 75–79 and ≥ 80 years) and region of England. Randomly permuted block sizes is used within strata.

### Intervention and comparator groups

**Fig. 2:**
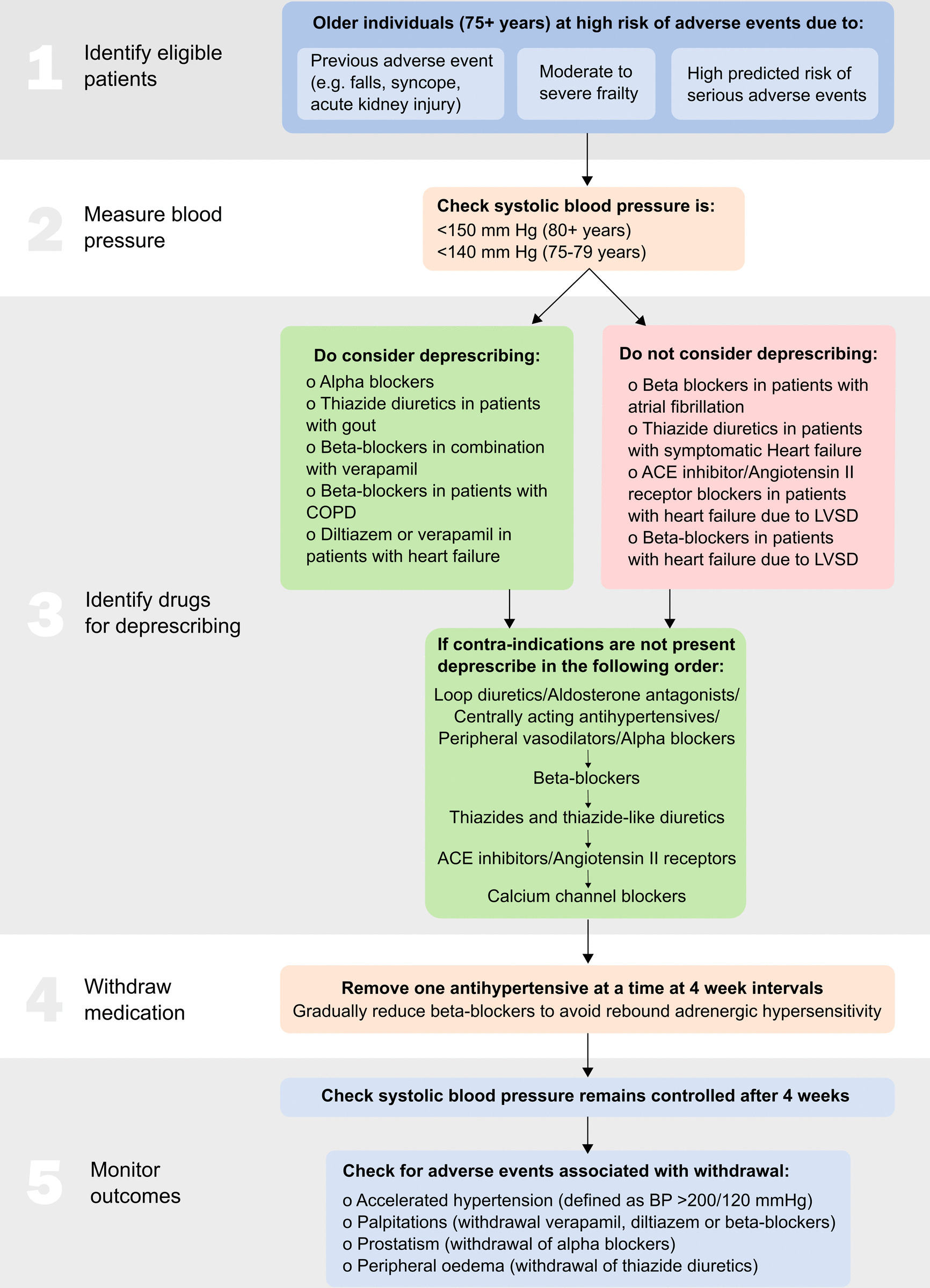
Antihypertensive deprescribing algorithm. ACE, angiotensin-converting enzyme; COPD, chronic obstructive pulmonary disease; LVSD, left ventricular systolic dysfunction; BP, blood pressure

The intervention is step-down antihypertensive medication reduction in comparison with usual care. Those in the intervention arm have antihypertensive medications removed one at a time under the supervision of the GP/prescriber, maintaining a systolic blood pressure of less than 140 mm Hg (in those aged 75–79 years) or 150 mm Hg (in those aged 80 + years). GPs/prescribers are given guidance on how to choose medications for deprescribing, outlined in Fig. 2, in line with approaches taken in previous studies [29, 30], but the final decisions on which drug to withdraw are left to them. This decision about which medications to remove is detailed in a medication reduction plan (prepared ahead of the baseline visit). Participants randomised to medication reduction will be informed which medication the GP has recommended for deprescribing and be asked to return it to their local pharmacy. There are no validated instruments for measuring compliance with medication reduction. GP prescribing data will be collected from electronic health records as a measure of compliance with the trial protocol. Those allocated to the control group receive usual clinical care, with no medication changes mandated. The trial uses an open-label design, so patients and practitioners are not blinded to the intervention or endpoints. Analysis of outcomes is blinded to the intervention allocation.

To maintain fidelity of the intervention, each medication is completely stopped, regardless of type or dose. Gradual dose reduction is not warranted, since any effects of withdrawing medication will be captured at the 4-week safety visit. The only exception to this is when withdrawing beta-blockers or clonidine, where the dose can be reduced first to avoid rebound adrenergic hypersensitivity.

### Follow-up

All participants (regardless of randomised group) are asked to return to their GP practice approximately 4 weeks following the baseline visit to have their blood pressure checked, and to report any adverse events that may have occurred. To ensure the safety of participants, blood pressure readings are taken in person. For those in the intervention arm whose blood pressure remains well controlled up to a target blood pressure of < 150 mmHg (or < 140 mmHg for those 75–79), the person performing the visit refers to the medication reduction plan and deprescribes a further antihypertensive if indicated. If another medication is deprescribed, participants are asked to attend a further safety visit 4 weeks later. This is repeated until there are no further antihypertensives to deprescribe, or until the GP/prescriber or participant feel it is inappropriate to deprescribe further. If participants’ blood pressure has risen above target at the safety visit, site staff are advised to invite them for another visit 1 week later to recheck their blood pressure. If the readings are still above threshold, further monitoring or re-prescribing of withdrawn medication is at the clinician’s discretion.

All follow-up after the safety visits are conducted remotely. Patient-reported outcomes are collected via questionnaires completed 1 year after participant’s baseline visit, which are either sent via email or post depending on individual participant preference. Clinical outcome data is extracted from electronic health records using three approaches. Firstly, primary care data is collected directly from participant’s records via a purpose-built database search run in each participating practice at the end of follow-up. Secondly, all participants are linked using unique trial identifiers, NHS number, and one other identifier (date of birth) to data held by NHS England. This includes Hospital Episode Statistics (the commissioning dataset that captures hospital activity such as diagnoses, procedures and therapies nationally) and Civil Registrations of Death, as provided by the Office of National Statistics. Thirdly, where possible and appropriate, primary care data is extracted remotely from the electronic health records of participants registered at primary care sites contributing to the Oxford Clinical Informatics Digital Hub (ORCHID).

### Safety reporting

Given the vulnerable nature of the population, adverse events are expected to be very common and usually unrelated to the intervention of interest. As a result, formal safety reporting is limited to those adverse events deemed related to antihypertensive medication reduction (adverse drug withdrawal events or ADWEs) or considered serious, as determined by the GP (or qualified and appropriately trained health professional responsible for hypertension care or medicines management). Adverse events of interest for this trial are those relating to fractures or falls, the circulatory system, kidney problems, electrolyte abnormalities, or hypotension/syncope. Adverse events occurring during the active follow-up period of the trial (1 year from randomisation for each participant) that are observed by the Investigator or reported by the participant or their carer are reported on the trial CRF, whether or not attributed to trial medication.

The following events are exempt from serious adverse event reporting procedures:

- Admission to hospital for routine, planned medical procedures or health assessment (e.g. hip replacement, routine transfusions).
- Admission to hospital or prolongation of hospitalisation for any reason other than for health purposes (e.g. prolongation of hospitalisation while appropriate social care is set up).
- Hospital visits for < 24 h that do not result in in-patient admission, unless considered an important medical event.

### Trial committees

The OPTIMISE2 Trial Management Group (TMG) is responsible for the monitoring of all aspects of the trial’s conduct and progress, and will ensure that the protocol is adhered to and that appropriate action is taken to safeguard participants and the quality of the trial itself. The TMG comprises individuals responsible for the trial’s day-to-day management (e.g. the Chief Investigator/Trial Lead, Trial Manager, Statistician, Data Manager) and will meet at least monthly throughout the course of the trial.

A Trial Steering Committee (TSC) provides overall supervision of the trial and ensures the trial is conducted in accordance with the principles of GCP. The TSC includes at least 75% of members who are independent of the investigators, including an independent chairperson. An independent Data Monitoring & Ethics Committee (DMEC) reports to and advises the TSC and includes two independent physicians and a statistician. The DMEC and TSC convene at 6-monthly intervals with sufficient time between each committee for the DMEC’s findings to be sent to the TSC ahead of their meeting. They will review the accruing trial and safety data to ensure trial site staff and participants are aware of any relevant safety information and to determine whether any reasons exist for the trial to be discontinued.

### Data management

The data management aspects of the trial are fully described in the Data Management Plan. This trial uses the RedCap eCRF system, where data are stored on secure servers with user access and logins controlled by our data manager. Access to the database is granted only after receipt of the database training log. Within the database, participants are identified only by a unique Trial ID, which is generated for every participant enrolled in the trial. The trial databases include secure login for staff at participating sites and facilities for manual entry of data and upload of files where appropriate. Single data entry is used in this trial, with automated checks built into the database that fire upon entry of data.

All data is stored within the database until all analyses have been completed, in compliance with the University of Oxford standard operating procedures for data retention. On completion of the trial and data cleaning, the trial documentation will be transferred to a secure, GCP compliant archiving facility, where it will be held for at least 5 years.

### Participant confidentiality

The trial complies with the UK General Data Protection Regulation (GDPR) and Data Protection Act 2018, which require data to be de-identified as soon as it is practical to do so. The processing of the personal data of participants is minimised by making use of a unique participant trial number only on all trial documents and any electronic database(s) (excluding consent forms). All documents are stored securely and only accessible by trial staff and authorised personnel. The trial staff safeguard the privacy of participants’ personal data.

### Sample size

The trial aims to randomise 3,014 participants and actively follow them up for 1 year to determine whether there is a non-inferior difference in death or first emergency hospital admission between treatment groups. This sample size gives 90% power to demonstrate a non-inferiority margin of 5%, at a 2.5% 1-sided level of significance, allowing for a 5% loss to follow-up.

This is based on a rate of emergency hospital admissions in the usual care group of 20.7% at 12 months, based on previously reported rates from HES data [31] inflated by 1% to take into account those people that die without a preceding emergency admission [32]. Due to the efficient trial design being employed, where most outcomes are measured using linked electronic health records, minimal loss to follow-up is expected. However, the sample size does include adjustment for up to 5% loss to follow-up, in the event that patients withdraw completely or records cannot be linked, based on the previous trial (where 1.4% of participants were lost to follow-up) [12].

### Statistical methods for primary and secondary outcomes

The primary analysis will be according to randomised treatment assignment and will include all randomised participants for whom data are available, as defined by protocol eligibility criteria. Deprescribing antihypertensive medication will be deemed non-inferior to usual care in terms of death or emergency hospital admission if the lower limit of a 95% confidence interval for the absolute risk difference between usual care and medication reduction groups is above − 5%, i.e. if the percentage of emergency hospitalisations/deaths in the deprescribing group is less than 5 percentage units higher than in the usual care group. This primary outcome will be analysed using a generalised linear mixed effects model. The response will be a binary indicator of whether the participant experienced death or emergency hospitalisation at 1 year. The model will include age and NHS England region as fixed effects and site as a random effect. Any covariates found to be predictive of missingness in the outcome, through fitting univariate logistic regression models, will be included in the model.

Generalised linear models adjusting for age as a fixed effect and site as a random effect will be used for secondary outcomes e.g. differences in the proportions of patients with serious falls, serious hypotension, syncope, fracture, hospitalisation/death, any non-serious adverse event, ADWE, all-cause death, major cardiovascular events, stroke, MI and perceived side effects to antihypertensive at 12 months, differences in mean change in treatment burden score, number of prescribed medications, number of prescribed antihypertensives, blood pressure, quality of life at 12 months.

### Health economic evaluation

The health economic evaluation aims to provide evidence on whether or not deprescribing of antihypertensives is cost-effective. Two forms of economic evaluation will be conducted: (i) a within-trial economic evaluation of deprescribing that mirrors the time horizon of the non-inferiority trial; and (ii) a decision-analytic modelling-based economic evaluation that will extend over a lifetime horizon. The economic assessment methods will adhere to the recommendations of the National Institute for Health and Care Excellence (NICE) Reference Case [33]. 

The within-trial economic evaluation will be conducted from the recommended NHS and personal social services perspective [33]. Primary research will be undertaken to estimate the cost of implementing deprescribing, including the costs associated with patient identification, monitoring activities and any follow-up/management. Broader resource utilisation is captured through two principal sources: (i) the electronic primary care health records and linked Hospital Episode Statistics; and (ii) Client Service Receipt Inventory questionnaires completed as described above at follow-up. Unit costs for resource inputs will largely be derived from national reference tariffs. Responses to participant EQ-5D-5L questionnaires completed at baseline and 12 months post-randomisation will be converted into health utilities using established utility algorithms for the purposes of quality-adjusted life year (QALY) estimation, with QALY profiles estimated using the trapezoid rule. Bivariate regression of costs and QALYs, with multiple imputation of missing data, will be conducted to generate within-trial estimates of incremental cost-effectiveness associated with deprescribing. Sensitivity analyses will be undertaken to assess the impact of areas of uncertainty surrounding components of the economic evaluation.

Decision-analytic modelling will be used to extrapolate the impact of deprescribing antihypertensives beyond the follow-up period of the main quantitative evaluation. The team will adapt the model used in the original OPTiMISE study [35] to estimate the impact of deprescribing on lifetime costs and QALYs, with cost-effectiveness expressed in terms of incremental cost per QALY gained. Multi-parameter uncertainty in the model will be addressed using probabilistic sensitivity analysis [36]. Sensitivity analyses will also examine the minimal level of adverse event risk required at baseline for deprescribing to be accepted as a cost-effective strategy.

## Discussion

Current clinical guidelines suggest that doctors take into account an individual’s frailty and multimorbidity when prescribing antihypertensive treatment in older adults [37] and consider the potential for medication withdrawal where blood pressure is low and frailty is high [38]. Currently, these recommendations are not evidence-based, with very few studies examining the effects of antihypertensive deprescribing in older people having been undertaken.

A recent Cochrane review found just six trials of antihypertensive deprescribing in patients aged 50 years or older (including 1073 participants) with a maximum follow-up of 56 weeks [39]. This review found no evidence of an effect of antihypertensive deprescribing on clinical outcomes; however, very few of the trials included in this review reported outcome events of interest, and therefore, it was underpowered to show any association with hospitalisation (19 events), mortality (18 events) or cardiovascular disease (3 events).

There are some ongoing trials, including the RETREAT-FRAIL trial, which is evaluating the long-term effects of deprescribing antihypertensive therapy in nursing home residents over the age of 80 with low blood pressure (< 130 mm Hg) treated with at least 2 antihypertensive drugs [40]. The RETREAT-FRAIL trial examines the effect of gradual reduction of antihypertensive treatment on mortality during a follow-up period of an average of 3 years. It will provide valuable information about the effects of antihypertensive deprescribing in a nursing home setting, in very frail older adults, where deprescribing may be undertaken in patients with limited life expectancy. In this setting, antihypertensive treatment may be considered at the very least futile, if not potentially harmful. However, these results may not be generalisable to older adults living independently in the community, where the balance of benefit to harm from antihypertensive treatment may be much more finely balanced. For this reason, the OPTIMISE2 trial is urgently needed to support clinical decision-making for such adults in primary care.

## Trial status

The trial is open to recruitment, having received full approvals on 18th September 2023. The first participant was randomised on 16th November 2023, and recruitment is planned to continue until at least the end of 2025. Current Protocol version 3.0 dated 19-Mar-2024. Any amendments to the protocol will be communicated to all relevant parties and trial sites.

## Dissemination policy

Results from this work will be communicated to key audiences through scientific journals, patient summaries and presentations at scientific meetings and community engagement events. Some time after the trial has finished, the results will be published on the trial website. Participants will be contacted to notify them that the results have been published and giving them the weblink to the results.

## Supplementary Information

Supplementary Material 1.

## Data Availability

Data sharing statement: Requests for sharing of de-identified individual participant data and a data dictionary defining each field in the set will be considered by the corresponding author.

## Abbreviations

ACE: Angiotensin-converting enzyme
ACEI: Angiotensin-converting enzyme inhibitors
ADR: Adverse drug reaction
AE: Adverse event
AR: Adverse reaction
ADWE: Adverse drug withdrawal event
CI: Chief Investigator
CPRD: Clinical Practice Research Datalink
CRF: Case report form
CRN: Clinical Research Network
CSR: Clinical study report
CT: Clinical trials
CTA: Clinical Trials Authorisation
CVD: Cardiovascular disease
DMEC: Data Monitoring and Ethics Committee
DSUR: Development Safety Update Report
eCRF: Electronic case report form
eFI: Electronic Frailty Index
EMIS: Egton Medical Information Systems
GCP: Good Clinical Practice
GP: General practitioner
HES: Hospital Episode Statistics
HRA: Health Research Authority
IB: Investigators Brochure
ICF: Informed Consent Form
ICH: International Conference on Harmonisation
ICMJE: International Committee of Medical Journal Editors
IMP: Investigational Medicinal Product
IRB: Independent Review Board
LVSD: Left ventricular systolic dysfunction
MHRA: Medicines and Healthcare products Regulatory Agency
mmHg: Millimetres of mercury
NHS: National Health Service
NICE: National Institute for Health and Care Excellence
NIHR: National Institute of Health Research
OPERAM: Optimizing Therapy to Prevent Avoidable Hospital Admissions in Multimorbid Older Adults
OPTiMISE: OPtimising Treatment for Mild Systolic hypertension in the Elderly
OPTIMISE2: Optimising Prescription of Treatment In older patients with Mild hypertension at Increased risk of Serious adverse Events
ORCHID: Oxford Royal College of General Practitioners Clinical Informatics Digital Hub
PI: Principal Investigator
PIL: Participant/Patient Information Leaflet
QALY: Quality-adjusted life year
QOF: Quality and Outcomes Framework
R&D: NHS Trust R&D Department
REC: Research Ethics Committee
RES: Research Ethics Service
RGEA: Research Governance, Ethics and Assurance
RSI: Reference Safety Information
SAE: Serious adverse event
SAP: Statistical analysis plan
SAR: Serious adverse reaction
SDV: Source Data Verification
SMPC: Summary of Medicinal Product Characteristics
SOP: Standard operating procedure
SSAR: Suspected serious adverse reaction
STRATIFY: STRAtifying Treatments In the multi-morbid Frail elderlY
SUSAR: Suspected unexpected serious adverse reactions
TMF: Trial Master File
TSC: Trial Steering Committee
